# Mental Health Therapy Protocols and eHealth Design: Focus Group Study

**DOI:** 10.2196/15568

**Published:** 2020-05-06

**Authors:** Marierose M M van Dooren, Valentijn Visch, Renske Spijkerman, Richard H M Goossens, Vincent M Hendriks

**Affiliations:** 1 Faculty of Industrial Design Engineering Delft University of Technology Delft Netherlands; 2 Parnassia Addiction Research Centre Brijder Addiction Care Parnassia Group The Hague Netherlands; 3 Department of Child and Adolescent Psychiatry Curium-Leiden University Medical Center Leiden Netherlands

**Keywords:** eHealth design, mental health care, personalization, protocol, youth addiction care

## Abstract

**Background:**

Electronic health (eHealth) programs are often based on protocols developed for the original face-to-face therapies. However, in practice, therapists and patients may not always follow the original therapy protocols. This form of personalization may also interfere with the intended implementation and effects of eHealth interventions if designers do not take these practices into account.

**Objective:**

The aim of this explorative study was to gain insights into the personalization practices of therapists and patients using cognitive behavioral therapy, one of the most commonly applied types of psychotherapy, in a youth addiction care center as a case context.

**Methods:**

Focus group discussions were conducted asking therapists and patients to estimate the extent to which a therapy protocol was followed and about the type and reasons for personalization of a given therapy protocol. A total of 7 focus group sessions were organized involving therapists and patients. We used a commonly applied protocol for cognitive behavioral therapy as a therapy protocol example in youth mental health care. The first focus group discussions aimed at assessing the extent to which patients (N=5) or therapists (N=6) adapted the protocol. The second focus group discussions aimed at estimating the extent to which the therapy protocol is applied and personalized based on findings from the first focus groups to gain further qualitative insight into the reasons for personalization with groups of therapists and patients together (N=7). Qualitative data were analyzed using thematic analysis.

**Results:**

Therapists used the protocol as a “toolbox” comprising different therapy tools, and personalized the protocol to enhance the therapeutic alliance and based on their therapy-provision experiences. Therapists estimated that they strictly follow 48% of the protocol, adapt 30%, and replace 22% by other nonprotocol therapeutic components. Patients personalized their own therapy to conform the assignments to their daily lives and routines, and to reduce their levels of stress and worry. Patients estimated that 29% of the provided therapy had been strictly followed by the therapist, 48% had been adjusted, and 23% had been replaced by other nonprotocol therapeutic components.

**Conclusions:**

A standard cognitive behavioral therapy protocol is not strictly and fully applied but is mainly personalized. Based on these results, the following recommendations for eHealth designers are proposed to enhance alignment of eHealth to therapeutic practice and implementation: (1) study and copy at least the applied parts of a protocol, (2) co-design eHealth with therapists and patients so they can allocate the components that should be open for user customization, and (3) investigate if components of the therapy protocol that are not applied should remain part of the eHealth applied. To best generate this information, we suggest that eHealth designers should collaborate with therapists, patients, protocol developers, and mental health care managers during the development process.

## Introduction

Adequate treatment is needed to reduce the risk of adolescents developing adverse consequences due to mental health disorders (eg, [1,2]). Therapy protocols contribute to the implementation of evidence-based therapeutic practice and help therapists to structure their face-to-face therapy sessions [3]. Although psychosocial therapies are effective in reducing psychiatric symptoms in adolescents with mental disorders, the available therapies show only modest effects and not all adolescents benefit [4,5]. The use of information and communication technologies in therapeutic practice, including electronic health (eHealth) interventions, in the delivery of (mental) health care [6] is a promising means to improve patient engagement and therapeutic effectiveness (eg, [7-9]).

The therapy protocols that form the basis for face-to-face therapies are typically used as a basis for the design of eHealth strategies [10]. Therapy protocols play a large role in the success of evidence-based therapies [11], and therefore their implementation is recommended as much as possible. However, both therapists and patients can personalize or only partly apply a therapy protocol in therapeutic practice (eg, [12-17]). For example, therapists might consider that following therapy protocols can be a hindrance to forming a strong therapeutic alliance [18]. Moreover, strict adherence to protocols may be perceived to come at the expense of building trust between a patient and therapist, which is an indicator for positive therapy outcomes by allowing them to work together in an effective way [19].

The gap between therapy protocols and their implementation in therapeutic practice has serious consequences for eHealth design. If the possibilities of personalization in therapeutic practices are not taken into account during the design stage, eHealth may not suit current therapy practice, thereby severely limiting its implementation. This can occur when eHealth does not suit how therapists use the therapy protocol or if therapists have negative expectations about the benefits of eHealth compared to face-to-face therapy [20-22]. Indeed, many eHealth interventions have failed to integrate personalization to the individual user in the design [23].

To align eHealth to the reality of therapeutic practice, it is important to understand the content of the existing therapy protocols and how they are applied in practice by both therapists and patients. Designers of eHealth can then use this information to ensure that eHealth matches the needs of therapeutic practice, consequently improving the quality and enhancing the implementation potential of the intervention. Toward this goal, the aim of this explorative study was to gain insight into personalization practices in a mental health care context and provide recommendations to eHealth designers on how they can best access and involve the need for protocol personalization in eHealth design. To achieve this, we examined therapists’ and patients’ perceptions of protocol application in a youth addiction treatment facility as a case study by generating both quantitative and qualitative data. First, we conducted focus groups to assess the extent to which therapists and patients personalized and applied a common therapeutic protocol in therapeutic practice. Second, we conducted focus groups with patients and therapists together to assess the degree to which they applied and personalized the therapy using the quantitative data generated from the first focus group as input for discussion.

## Methods

### Therapy Protocol

The commonly applied protocol for cognitive behavioral therapy (CBT) in adolescent addiction care was used as a case protocol [24]. The protocol consists of 9 sessions, followed by 4 “sessions of choice” (selected from 7 optional sessions in consensus with patients). In each session, patients set specific short-term goals with regard to the therapeutic homework. Part of the therapy protocol is a therapy workbook that patients can bring home and to therapy sessions. The activities described in the workbook correspond to the content of therapy sessions.

### Procedure

We conducted semistructured focus group sessions in two phases at two locations of one large outpatient treatment facility center for adolescent addiction care in the Netherlands (see Table 1). The aim of the first phase was to investigate therapists’ and patients’ estimations of the amount and type of protocol personalization.

Therapists estimated how much of their therapy consisted of a strictly followed and adapted therapy protocol and patients indicated how much of the therapy provided by their therapist they strictly followed and adapted. Of note, the patient could be receiving a personalized therapy protocol in practice. Both the therapists and patients also indicated how much other (nonprotocol) therapeutic components were added in practice, which were represented by percentages for a total of 100% (ie, the whole therapy). The second phase involved an independent group of therapists and patients who did not participate in the first phase, in which the results of the first phase were applied to the discussion to gain insight into the reasons for personalization. Participants were brought together with a moderator (the first author MvD) for a discussion lasting 1 hour. Before starting the group discussions, the participants provided informed consent and the concept of personalization was explained (ie, changing a designed end product such as a therapy protocol to match the needs and capacities of the end user and enhance effectivity of the product [25]).

**Table 1:** Setup of the focus group sessions with therapists and patients on protocol application and personalization.

Goal	Phase	Participants
Generate information with separate groups of either therapists or patients about how much of their therapy consisted of a strictly followed therapy protocol, adapted therapy protocol, and added therapeutic parts.	1	Group discussion with therapists (location A N=3, location B N=3), group discussion with patients (location A N=2, location B N=2), and interview with a patient (N=1, location B).
Joint evaluation of the results from Phase 1 with combined groups of both patients and therapists.	2	One mixed group discussion with a therapist and two patients at location A and one mixed group discussion with two therapists and two patients at location B.

### Participants

We invited experienced therapists who received training in the CBT protocol to participate in focus group sessions. Patients, who were at least 18 years old and receiving CBT, were recruited by their therapists to participate in the study. Therapists received an information leaflet to inform their patients about the study. A therapist informed us if a patient wanted to participate. In turn, we contacted the patient to schedule an appointment for the focus group discussion. In the first phase, 6 therapists (3 women, 3 men) and 5 patients (1 woman, 4 men) participated. In the second phase, 3 therapists (1 man, 2 women) and 4 patients (1 woman, 3 men) participated. All interviews took place at the youth mental health care facility.

### Data Analysis

The data are presented according to the standards of reporting qualitative research proposed by O’Brien et al [26]. We used thematic analysis instead of grounded theory to analyze the data. With grounded theory, the goal is to generate an exploratory and overarching framework or theory [27], which was not the goal of this study. Alternatively, with thematic analysis, the themes are derived from the data [28-30], which can offer direct guidance for eHealth designers. We focused on the phases described by Braun and Clarke [28] for data analysis. The interviewer (MvD) is a PhD candidate with two masters degrees in clinical and health psychology. She is therefore qualified in conducting qualitative interviews, and did not have any assumptions or prior relationships with the participants before the discussions. All interviews took place at the youth mental health care facilities of the therapists and patients. Experienced therapists were invited to participate in the study. They had to be trained in the new CBT protocol that was the focus of the study. Patients who had received CBT were informed about and invited to participate in the focus group discussions by their therapists. In this way, more patients were informed about the study, which enhanced the chance that more patients would be willing to participate. After the patients provided consent to participate, the researcher contacted the patient by phone to ensure that they clearly understood the study and to make an appointment for the focus group discussion.

We received formal ethics approval from the Human Research Ethics Committee of the Delft University of Technology in the Netherlands. All discussions were recorded with an audio recording device after receiving verbal consent from participants. Quantitative data were saved with only a link to the type of participant (ie, therapist or patient). All focus group sessions took 1 hour each and were audio recorded and transcribed by one author (MvD). Interview guides were used during the discussions, and field notes were taken both during and after the discussions.

After transcribing the data, all recordings were checked again to ensure the accuracy of the transcripts in line with the recommendations of O’Brien et al [26]. All of the transcripts were then reviewed multiple times before coding the data by the same author (MvD). This ensured that the themes generated from the codes were not based on only a few examples. Similar themes were grouped together into higher-level themes. When analyzing the data, the themes were linked to each other, ensuring a coherent story. Sufficient time was allocated to analyze the data adequately.

The fourth topic of O’Brien et al [26] focuses on the results (topics 16 and 17) that are described in the following section. Supportive quotes were chosen to substantiate analytic findings. This was followed by the fifth topic that describes the discussion section (topics 18 and 19) and the “other” topic that deals with conflicts of interests and funding (topics 20 and 21).

## Results

### Phase 1: Focus Group Sessions With Therapists

Therapists indicated that they strictly applied 30%-75% (mean 48%) of the therapy protocol and adapted between 10% and 50% (mean 30%) of the therapy protocol. They further reported adding 10%–33% (mean 22%) nonprotocol-related therapeutic components. The percentages of one therapist were excluded because the percentages of strict application and personalization overlapped.

We first scrutinized the quotes several times and generated codes from the quotes that focused on reasons for therapists choosing to personalize the therapy protocol. These codes referred to therapists who personalized the protocol based on a patient’s needs (“Tweaking works the best, adapting [the therapy] to where the patient is”: Therapist 1A); what they thought would be more beneficial for the patient (“It is more related to whether they have stopped [using substances] than focusing on cravings. Also, if they already went to therapy before, elements considered to be repetitive are removed”: Therapist 2A); and because therapists were aware of other therapy protocols that could help patients with different problems at the same time (“I also give group therapy, and some elements that I notice work [during group therapy] I also use during individual therapy”: Therapist 1B). In addition, the codes reflected that therapists personalized the protocol to enhance the therapeutic alliance (*“*Much is related to the connection, the therapeutic alliance is important so I invest a lot of time in building one”: Therapist 1B). Further analysis of the codes resulted in higher-level themes.

The main theme derived from the codes of the therapists was that they used the protocol as a “toolbox” (ie, a bundle of therapy tools that they could choose from). The code that did not fit this main theme focused on adding elements from other therapy protocols. However, all therapists mentioned that they did not apply the order of the therapy protocol in a strict manner. The protocol was not considered as a step-by-step manual but rather as a manual comprising all possible interventions: “I do not use the CBT toolbox as a step-by-step manual but I can choose interventions from the toolbox that I find relevant” (Therapist 1B). Three subthemes were derived: therapists who personalized based on what they thought their patient needed, on their own therapy-provision experiences, or because they thought it enhanced the therapeutic alliance.

The first subtheme under the grand toolbox theme consisted of therapists who personalized the therapy protocol based on what they thought their patient needed. They considered that by adapting the therapy, their patients would be better prepared to handle specific situations. This was influenced by the (possibly difficult) situations that patients experienced prior to the therapy session (eg, had an argument with their parents), how the motivation of patients could change their behavior, and if patients understood all elements of the therapy protocol. For example, therapists tried to enhance the trust of patients by ensuring them that they could achieve the goals they set or by mainly focusing on the homework that a patient did well instead of focusing on the homework that a patient did not do well:

What is important for patients, such as dealing with social pressure. In general, I follow the therapy protocol but if you notice that patients have difficulties with it [social pressure] you focus on that.

Therapist 3A

The second subtheme to the grand toolbox theme consisted of therapists who personalized the therapy protocol based on their own therapy-providing experiences. During the discussions, they commented that they might not apply or only partly apply the workbook to prevent their patients from experiencing feelings of failure, since patients generally forget to bring it to therapy or fill in the homework assignments. Therapists thought that not applying the workbook prevented their patients from experiencing feelings of failure:

I always estimate if they [the patient] are the type of person that can do homework at home, if they [the patient] are someone who will really do it [the homework], you want to prevent experiences of failure.

Therapist 3B

In addition, more experienced therapists have more knowledge of and experience with other types of therapy protocols. Therefore, more experienced therapists tend to apply elements from other therapy protocols during therapy more often compared to less experienced therapists.

The third subtheme to the grand toolbox theme consisted of therapists who personalized the therapy protocol because they thought it would enhance the therapeutic alliance:

It depends on the connection [between me and the patient]. The therapeutic alliance is important, on which I spend a lot of time.

Therapist 1B

They would try to work on the bond with a patient by focusing more on the positive steps a patient made rather than focusing on what a patient did not do. In addition, this was expected to enhance the motivation of patients to continue with therapy and try to achieve the tasks they agreed on.

### Phase 1: Focus Group Sessions With Patients

Patients indicated that they strictly applied 12%-65% (mean 29%) of the therapy provided by their therapist, adapted between 9%-64% (mean 48%), and added between 18%-26% (mean 23%). The percentages of one patient were excluded, because the percentages of strict application and personalization overlapped.

After scrutinizing the quotes several times, we generated codes that focused on reasons for patients choosing to personalize their therapy. These codes referred to patients who personalized how they achieved their homework because they preferred to personalize the tasks, “Actually I try to think of some rules for myself” (Patient 1B), and because they were somewhat careless and forgot to complete their homework, “It is quite hard to keep up with it and it is not really in my routine, like brushing my teeth” (Patient 2A). In addition, the personalization of patients was influenced by the connection they had with their therapist: “The connection you have with your therapist influences how well therapy works” (Patient 2A). We went through the codes again, resulting in higher-level themes.

The main theme that was derived from the codes of the patients was that they personalized the therapy based on their own situation. The code that did not match the main theme focused on personalization of therapy by the therapists. Even though therapists and patients decided on the homework the patient would work on together, all patients mentioned that they personalized their homework. Two subthemes were derived: personalization to better match therapy with the daily life of the patient, and personalization that was influenced by the varying motivation of patients.

The first subtheme to the grand own situation theme focused on patients who mentioned that they personalized their therapy to better match the therapy with their daily lives. That is, they personalized the therapy to achieve a better match with their own situation, personality, and preferences. This helped to reduce their feelings of stress and worry:

I always change it [doing the homework assignments] a little bit so that it is in line with my personality and how I want to be seen by others.

Patient 2A

…It is the intention [to do the workbook assignments], but I don’t do it. I would rather talk about it [cravings] than write these experiences down.

Patient 1B

The second subtheme to the grand own situation theme consisted of patients who mentioned that the extent of their personalization was influenced by their varying motivation. In some cases, they simply did not want to or forgot to do the homework assignments. In addition, a relapse could influence the motivation to continue therapy in either a positive or a negative way: “Sometimes, I just do not feel like doing it [workbook assignments] and I just do not do it” (Patient 1A). One patient said that doing the workbook assignments for a longer period of time helped him to generate insights into his triggers for cravings. The therapeutic alliance influenced their motivation, mainly because a therapist would put things into perspective (including when a patient had a relapse).

**Figure 1:**
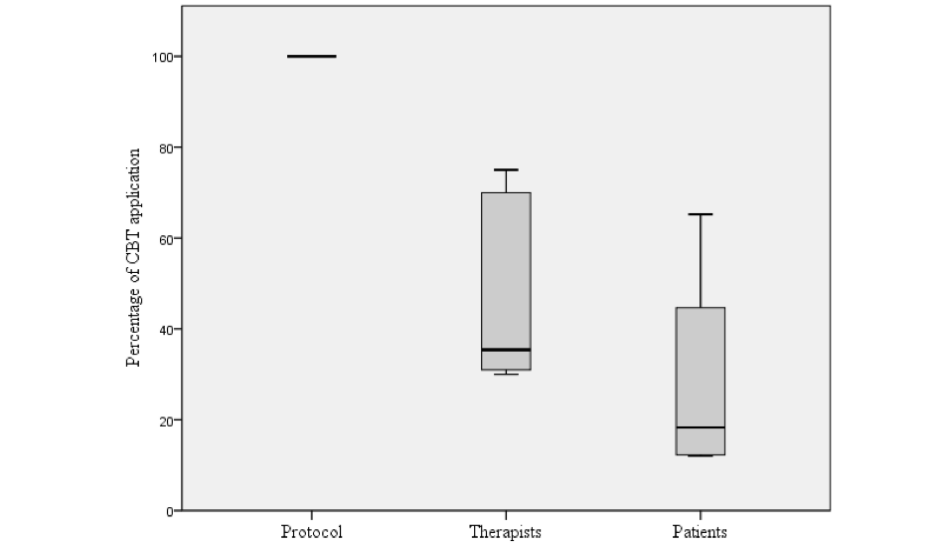
Range and median percentage of cognitive behavioral therapy application by therapists and patients.

Along with the thematic analysis, we analyzed the quantitative data of the patients and therapists. All of the percentages related to therapy protocol application and personalization by therapist and patients are combined in Figure 1, and these data were used in the second phase of the focus group discussions with a separate group of patients and therapists together.

### Phase 2: Focus Group Sessions With Both Patients and Therapists

We first analyzed the quotes that focused on reasons for patients and therapists to personalize the therapy protocol and generated codes from these quotes. The codes of the therapists referred to personalization aimed at maintaining or enhancing the motivation of patients, to work on a connection of trust with their patient, and to align the therapy to the problem of the patient. The codes of the patients referred to personalization by discussing what was happening in their life during a therapy session, and personalization of homework based on how they felt during therapy and at home. Further analysis of the codes resulted in higher-level themes.

Since therapists reported that they always personalize the therapy to some extent, most therapists and some patients had expected more personalization. One therapist thought that therapists could also have interpreted a strict therapy protocol application from the guideline, meaning that therapists did not apply the detailed and precise content of the therapy protocol sessions but rather used the content to assist them in making decisions about which elements of the therapy protocol sessions would be most appropriate for a given case: “I think that the therapists do follow the therapy protocol as a guideline, but that they noted this [strict application] down as applying it in an unchanged way” (Therapist 5B). Other themes derived from the codes in the second phase focused on enhancing the therapeutic alliance and on personalization based on the experience of therapists.

The first main theme of therapists derived from the quotes focused on enhancing the therapeutic alliance: “Aligning to the need of the other [the patient] and small talk [with the patient] contributes to the personal connection with a therapist, which contributes to a more personal relationship that is needed to create openness and allowing a patient to accept help from a therapist” (Therapist 4B). Fostering an alliance was seen as crucial in order for a patient to trust the therapist and work together to solve the problem of the patient. Two subthemes were derived: personalization based on the individual situation of a patient and keeping or enhancing the motivation of a patient.

The first subtheme to the grand therapeutic alliance theme focused on the individual situation of a patient. In general, therapists first focus on the individual situation of a patient, followed by the relevant therapy protocol session that best suits the situation. They could also apply elements from different therapy protocols when a patient had other psychological problems. In this way, patients were helped with all of their problems at the same time: “The patients often have multiple problems, so you have anxiety and mood protocols, or other ones” (Therapist 4B).

The second subtheme to the grand therapeutic alliance theme focused on keeping or enhancing the motivation of patients. They either did or did not apply the therapy workbook, mainly to prevent experiences of failure and maintain motivation to adhere to therapy if a patient forgets the workbook: “You also have to prevent that it [filling in the workbook] becomes a failing experience…they can think, well if I can’t even do that well…” (Therapist 4B). In addition, therapists applied motivational interviewing to enhance the motivation of patients: “It is part of the attitude as a therapist that you are empathetic, you listen and align” (Therapist 4B).

The therapy protocol helped therapists to structure the therapy, but therapists differed in their opinion regarding protocol application. One therapist from location A followed the therapy protocol as strictly as possible, whereas another therapist from location B only used the therapy protocol to guide the therapy sessions. The therapist from location A mentioned that the therapy protocols helped to provide guidance to the therapy sessions, whereas the therapist from location B found it more important to focus on the situation of a patient.

The second grand theme of the therapists focused on the experience of therapists that influenced the amount of personalization. More experienced therapists often have experience with different therapy protocols, since therapy protocols often change or improve over time. This increased their knowledge, preferences, and possibilities to personalize therapy protocols compared to less experienced therapists. Therefore, two therapists mentioned that the experience of therapists could also influence therapy protocol application and personalization.

Based on the quotes of the patients, we derived two main themes. The first grand theme focused on personalization based on their own personal situation. They personalized their homework based on possible relapses and how they felt:

I had to do exposure exercises once a day. But if I do not feel well, it does not work and I’m not going to let myself feel worse by doing another exercise.

Patient 4B

The second grand theme focused on personalization based on the personal preferences of patients. The strategies applied to work on their therapy and prepare for a therapy session differed among patients based on their personal preferences, such as by shutting down the mobile phone when starting therapy or working on assignments on a computer instead of in the workbook. The main themes and subthemes emerging from both phases of the discussion groups are summarized in Table 2.

**Table 2:** Themes and subthemes of the focus group discussions with therapists and patients.

Participant	Main theme	Subthemes
Therapists: Phase 1	Use protocol as a toolbox	Personalization based on patient needs; personalization based on own therapy-provision experiences; personalization to enhance therapeutic alliance
Patients: Phase 1	Personalization based on own situation	Personalization to better match therapy with daily lives; personalization influenced by varying motivation
Therapists: Phase 2	Personalization to enhance therapeutic alliance; personalization based on experience	Personalization based on the individual situation of a patient; personalization to maintain or enhance the motivation of patient
Patients: Phase 2	Personalization based on personal situation; personalization based on personal preferences	None

## Discussion

### Principal Findings

Existing research focusing on the effect of eHealth in mental health care suggests overall small to medium effect sizes [7,8,31-33]. Moreover, research suggests that combining eHealth with therapist contact (ie, blended eHealth) is more effective compared to fully online eHealth without therapist contact [9,34]. One main contributor to the effectiveness of eHealth is that it can extend the reach of psychological therapy beyond the clinical setting, as the technologies can be used anytime and anywhere [35,36]. eHealth designers typically use the therapy protocols of evidence-based face-to-face therapies as a basis for the design of eHealth. However, not all parts of therapy protocols are always applied in therapeutic practice [16,37]. If eHealth designers do not take this into account, the designed eHealth might not optimally fit the existing therapeutic practice, which will consequently impede implementation and motivation to adopt the eHealth by both therapists and patients. In the present study, we analyzed the proportion, type, and reasons for personalization of a given therapy protocol by therapists and patients in focus group studies.

The results showed that the therapy protocol is not fully applied in clinical practice but is also personalized (see Table 2), which is in line with previous studies [13,38]. The available therapy protocol is thus only one factor considered in a therapeutic process. Other factors that influence the therapeutic process are the personalization practices of therapists based on the needs of a patient, motivation of a patient, therapy-provision experiences of therapists, and the therapeutic alliance between the therapist and patient. Therapists estimated that they only strictly followed 48% of the protocol, adapted 30% of the protocol, and replaced 22% of the protocol by other nonprotocol therapeutic components such as other therapy protocol elements. Other personalization practices that influence the amount of therapy protocol application is personalization of patients to better match the therapy with their daily lives, personal situations, and preferences, which was also influenced by their varying motivation. Patients estimated that they strictly followed 29% of the therapy assigned, 48% of which was adapted, and they estimated that they replaced 23% of the therapy by other nontherapeutic elements.

It is important to mention the clear difference in personalization between therapists and patients. The estimations of patients and therapists regarding their amount of personalization are not only different because they may personalize less or more but also because of their own share in the personalization process. Therapists already personalize a therapy protocol, and their patients further personalize the elements provided according to their daily lives. Moreover, therapists are aware of the entire content of the therapy protocol, whereas patients are not. Since therapists provide the patient with a partly personalized therapy, patients can never fully know the entire possible content of a therapy protocol and have less personalization options of the standard therapy protocol. For example, therapists often mentioned that they did not use the therapy workbook to avoid patients from experiencing feelings of failure if they either did not do the homework assignments or forgot to bring the workbook to therapy. However, by doing so, they also prevented patients from trying to execute the homework assignments in their workbook. Moreover, personalization by therapists can have both positive, neutral, and negative effects [39-42]. For example, the elements that are personalized by a therapist or how a therapist personalizes specific protocol elements may not match with the preferences of a patient. This may influence the alignment of the therapy to a patient and may possibly lower motivation of a patient to adhere to the therapy. In general, most therapists in the second phase had expected that therapists would personalize more than suggested by the estimated percentages of protocol application from therapists in the first phase. A previous study that only focused on personalization by therapists found that therapists personalize more than our results suggest [43]. A possible explanation for this difference is that the previous study aimed at assessing all types of activities in the general psychotherapeutic practice of eating disorders, whereas we focused specifically on the personalization practices of both patients and therapists using a CBT protocol in youth addiction care as a case protocol.

### Implications and Recommendations

The results of our study have important implications for eHealth clients and eHealth developers by providing insight into the protocol elements in eHealth that should and should not be open for personalization to facilitate implementation and patient engagement. Designers can implement the personalization practices by focusing on the function that personalization has in therapeutic practice (ie, enhancing the motivation of patients to adhere to the therapy). However, since personalization may have both positive and negative therapeutic effects, it is important to know what elements are crucial to apply in practice to enhance therapeutic effects. This is particularly relevant since design can influence and enhance motivation to adhere to or execute specific behaviors. One such example is the application of motivating elements from entertainment games (also called “gamification”). Gamification design has shown potential in health care, and in mental health care in particular [44-46], such as by improving healthy behaviors [46-57]. Based on the results of this study, it is recommended that eHealth designers: (1) study and copy at least the actual applied parts of a therapy protocol in eHealth, (2) co-design eHealth with therapists and patients so they can allocate the components that should be open for user customization, and (3) investigate if components of the therapy protocol that are not actually applied by therapists or patients should remain part of the eHealth. Without such considerations, implementation would be negatively impacted owing to a mismatch from the habits of therapists [20] or the complexity of mental problems that patients experience [58]. In addition, validation studies of therapy protocols should focus on the actual application of these protocols in therapeutic practice, which can be considered to be generally overestimated [18,59,60]. This may in turn overestimate the benefit of therapy protocols to therapeutic effects. Below, we elaborate on these three recommendations.

With regard to the first recommendation, our study showed that therapists and patients do not fully apply the therapy protocol. This information should be generated and implemented in the second product design phase of a personalized design process [25]. In this phase, stakeholders such as therapists, patients, and protocol developers can be involved to ensure that the design of the product is suitable to support the user during therapy, that it is technically possible to use during therapy, and that the eHealth design suits the therapeutic practice of a treatment center. In this phase, the information of the applied therapy protocol elements by therapists and patients is generated so that eHealth designers can at least copy these components in the eHealth. One method of generating this information is to record therapy sessions of patients with therapists. Therapy protocol developers can then listen to these recordings and rate the parts of a therapy protocol that are applied in therapeutic practice.

As a second recommendation, the results of our study showed that therapists and patients personalized the therapy protocol by adjusting specific protocol components and adding other (nonprotocol) therapeutic components. Understanding why and how therapists and patients personalize a therapeutic protocol is important information for eHealth designers to select the components of eHealth that should be open to personalization for therapists and patients. This information can be generated and implemented in the last tailoring phase of a personalized design process [25] with patients and for more and less experienced therapists. In this phase, the designed product is tailored to the individual user using two main types of tailoring: user-controlled customization and use-dependent adaptation. With user-controlled customization, a user can tailor a product themselves according to their own preferences and needs. Patients noted that they personalized the therapy based on their own personal situation and personal preferences; thus, it is also important to give them the opportunity to do so when using an eHealth product. Therapists mentioned that they personalize a therapy protocol based on the patient situation or their therapeutic experiences. By providing therapists the possibility to tailor the elements in eHealth, they can choose whether or not to use these elements during therapy with a specific patient, while not being forced to use all elements of the eHealth app. With use-dependent adaptation, a product automatically adapts itself to the user, for example by not showing specific parts of a therapy protocol if the therapist always skips these in therapeutic practice or by tailoring the moment reminder popups to a patient who typically experiences cravings after dinner.

As a third recommendation, eHealth designers should investigate if there are components of the therapy protocol that are not actually applied by therapists or patients but should be part of the eHealth since they are crucial for the effect of therapy. The eHealth designer can generate this information by interviewing therapy protocol developers about the crucial therapy protocol components. This information can be generated by involving stakeholders in the second product design phase of a personalized design process [25]. One approach would be to allow the therapist to use the eHealth app as a toolbox, which can ensure that crucial elements are not too easily personalized or skipped.

### Limitations

Our study has two main limitations. The first concerns asking therapists and patients to quantify their own behavior, which may be challenging. Previous research also found that therapists overestimated the extent of therapy protocol application [61] or that self-reporting had very poor reliability [62]. For example, not all respondents understood the assignment, as the indicated percentages of strict therapy protocol application of a patient and therapist overlapped with their other percentages. This overlapping is impossible, as one cannot both strictly follow and change a therapy protocol at the same time. However, asking therapists and patients to quantify their own behavior may still be a suitable technique when asking them only to estimate the amount of therapy protocol application and personalization they adopt. Accordingly, this approach is considered suitable to generate first insights, but results cannot be solely based on this technique. A second limitation is that we did not take the therapeutic experience of the therapists and severity of the patients’ conditions into account [63]. Compared to less experienced therapists, more experienced therapists generally have more experience with other therapy protocols, which may influence their personalization practices. In addition, it is possible that the severity of a patient’s condition could have influenced recruitment and results. Another limitation is that this study was conducted with a limited number of participants, which might have enhanced the possible influence of individual preferences regarding protocol application and personalization on the results [64]. Future research should take this into account, such as by conducting the study with a larger sample size while taking into account these background variables. In addition, future designs of a toolkit should consider involving actual eHealth designers, eHealth design employers, and researchers. This is important since the toolkit may otherwise not correspond with current practices of target groups, which would negatively influence its implementation.

### Conclusions

To optimize eHealth implementation, our study indicates that eHealth designers should have information as to which therapeutic components should be duplicated, which components should be open to personalization possibilities, and which components that are not applied in practice should remain part of the eHealth design. To generate this information, we suggest that eHealth designers collaborate with therapists, patients, protocol developers, and mental health care managers during the design process of eHealth [25]. Not involving all of these stakeholders increases the risk that the designed eHealth might not optimally fit the therapeutic practice, which would impede implementation. For example, therapy protocol designers typically know what protocol components are crucial for the therapeutic effect but do not know how the protocols are applied and personalized in therapeutic practice. Personalization practices can be implemented by actively co-designing with patients and therapists with different levels of experience to ensure that the eHealth is aligned to their preferences and capacities. Based on the present research, we expect that the implementation of eHealth can be facilitated when stakeholder representatives (eg, patients, therapists, protocol developers, and mental health care managers) are collectively involved in the design process by providing the eHealth developer with their needs and demands of therapy protocol application and personalization.

## Abbreviations

CBT: cognitive behavioral therapy
eHealth: electronic health
